# Self-Destroyers

**Published:** 1882-03

**Authors:** 


### SELF-DESTROYERS.

According to the present generally received views of preparatory professional requirements, the student who has his diploma, the pulpit, or the bar in view, has no time for other than studies which qualify him directly for graduation in the university or the seminary; in fact, so many studies are compressed in such a comparatively short period, that there is not even time for a young gentleman of medium abilities to fully master the most essential elements, or if he does, it is from such close and incessant application that often, with commencement-day, he dies! or if he survives its reaction, his licensure is too frequently clouded and then closed forever, by the stealthy poison of a disease instilled during the diplomatic race. Many a reader of mine will be stricken with the painful remembrance of cases like these My mind has been painfully impressed, almost daily, with the conviction that the most useful and efficient men in the community are often lost to society, the church, and the world, from a remarkable ignorance of some of the simplest laws of their being. This is not to be wondered at; for, from the nursery, through the primary schools, the academy, the college, the seminary, to the study, not one single lesson is given how to save from a premature death the man who has prepared himself to act in the great drama of the world, by the expenditure of thousands of money, and a score of years of incessant and painful labor, and study, and research.

The design of this Journal is strictly practical; practical in the every-day sense of the word: its teachings will accompany the reader in nearly all the occupations of life; every hour of the day will afford him opportunities of carrying out its principles, not as a weary, fretting task, but in the way of an intelligent and pleasurable observation. The accomplished geologist, while travelling over barren wastes and rocky hills to explore some distant golden district, can make every pebble and every lump of earth minister to his instruction and amusement, without its at all interfering with the main object of his journey: just so may a man's mind be so well stored with intelligent information on the subject of health, and the general laws of life, that observations may be made on these every hour of his waking existence almost, with pleasure and with profit, without at all interfering with the main business of life, and also without having any undesirable influence on mind or body; for he may do this without being forever engaged in looking out for symptoms, aggravating those present or imagining those which are not. In short, the Journal will embrace whatever the Editor thinks will tend to the convenience, comfort, health, and perfection of the physical man.

It is not contemplated to issue a series of prose essays on "*Physiology*" or "*Hygiene*," nor to enter into technical and learned disquisitions on the chemical analysis of food, the philosophy of cell life and development, nor indeed any systematic expositions; but simply in short articles, in plain English, to treat on such subjects as may present themselves from time to time, calculated to bear upon the great points.

How to determine disease in its very first approach.

How to arrest it at once by natural agencies.

How to live so as to prevent sickness.

How to eat.

How to sleep.

How to exercise.

How to dress.

How to walk.

How persons become dyspeptic.

How and why the health of the young so often begins to fail while pursuing an education.

How they may so conduct their studies as to preserve the health, and yet accomplish in the course of the year a larger amount of mental and literary labor.

If an essay is admitted, it will be, among others, from eminent practical dentists, as to the best means of preserving the teeth perfect to the close of life, as perfect teeth are essential to a distinct enunciation, which is of indispensable importance to public men, whether professional or literary.

I will endeavor to make this publication such a one as will be proper for the youth of both sexes to read with increasing interest and attention, so as to induce them early, while the constitution is yet vigorous and unimpaired, so to study the nature and effects, on the human frame, of food, clothing, air, exercise, cheerfulness, system, industry, profitable and interesting employment, as will be an effectual guard against those negligences and indiscretions which so often lay the foundation for an early death

### SORES.

Sometime ago, a little child had a pimple on its breast, which became a little sore, an amateur doctor advised a "simple" remedy to be applied to it, which was done, neither the advice nor the remedy cost anything, except the child's life in forty-eight hours.

A gentleman had a small running sore on the top of his foot; he was anxious to have it "cured up," we advised him to keep it running, but that was troublesome, and it was healed up, in a short time, a cold set in, and he died of consumption, at the end of a year.

The son of a merchant wanted to reduce a swelling in the side, which began to "run;" general remedies were proposed, with advice to let the "sore" alone. This did not suit his views, so he had it healed up, and died of consumption within two years.

A lady had a sore on the leg: it interfered with her walking, she was impatient to have it "cured up," but was advised as to the consequences, but this was disregarded. The sore was healed up, and she died of consumption.

A lady aged seventy had a sore leg, and was extremely anxious to have it healed up. She was advised by all means to make no application to it, but merely to keep it under control by general remedies. An old woman was applied to, and with various salves a wonderful "cure" was the result. Within a few weeks the lady took dinner in usual health, and died in an hour.

A lady aged sixty six had a long continued violent and painful cough, a "running" took place under the toe nail, she feared mortification, and was anxious to have it healed up. She was told that it was the best thing that could have happened to her, inasmuch as it would probably cure her cough, and add years to her life, while by improving the general health the running might slowly dry up of itself, all which proved to be so, and now in her seventy-fourth year, she has better health than half our women at forty.

Our object in stating these facts which have occured in our own experience, within a comparatively short time, is to impress upon the reader's mind, the signal danger of tampering with sores, especially such as are sometimes called "old sores,"

for they are the outlet to disease, and if injudiciously closed, what they would have discharged, will be thrown in upon more vital internal organs, causing apoplexy, consumption or fatal congestions, as certainly as the boiler of a locomotive will be shivered to atoms, if the fire is continued, and all escape of steam is prevented. In all cases of old sores, apply to a physician of age and experience. If that is not practicable, the safest and best plan is first, to diminish the amount of food eaten each day, one half, and keep the parts in a cleanly condition, by washing them twice a day in soft, milk-warm water, until relief is given.

